# Quantitative evaluation of abnormal finger movements in myelopathy hand during the grip and release test using gyro sensors

**DOI:** 10.1371/journal.pone.0258808

**Published:** 2021-10-20

**Authors:** Shota Date, Kazuyoshi Nakanishi, Yasushi Fujiwara, Kiyotaka Yamada, Naosuke Kamei, Hiroshi Kurumadani, Manabu Yoshimura, Akio Ueda, Yosuke Ishii, Ryo Ohta, Shinji Kotaka, Yuji Tsuchikawa, Toshio Nakamae, Masakazu Ishikawa, Ken Hirao, Yoshinori Fujimoto, Nobuo Adachi, Toru Sunagawa

**Affiliations:** 1 Laboratory of Analysis and Control of Upper Extremity Function, Graduate School of Biomedical and Health Sciences, Hiroshima University, Hiroshima, Japan; 2 Department of Orthopedic Surgery, Nihon University Hospital, Tokyo, Japan; 3 Department of Orthopedic Surgery, Hiroshima City Asa Citizens Hospital, Hiroshima, Japan; 4 Department of Orthopaedic Surgery, JA Hiroshima General Hospital, Hiroshima, Japan; 5 Department of Orthopaedic Surgery, Hiroshima University Hospital, Hiroshima, Japan; 6 Department of Orthopedic Surgery, Hirao Clinic, Hiroshima, Japan; Tokai University, JAPAN

## Abstract

Previous studies have reported qualitative characteristics of myelopathy hand, but few studies have reported quantitative kinematic parameters of this condition. Our purpose of this study was to quantitatively evaluate the abnormal finger movements in patients with cervical compressive myelopathy (CCM) (termed myelopathy hand) and to understand the characteristics of myelopathy hand during the grip and release test (GRT) using gyro sensors. Sixty patients with CCM (severe: n = 30; mild-to-moderate: n = 30) and sixty healthy adults (age-matched control: n = 30; young control: n = 30) were included in this study. All participants performed the GRT. The index and little fingers’ and the wrist’s movements during the GRT were recorded using three gyro sensors. The number of cycles, switching time-delay, time per cycle, and peak angular velocity were calculated and compared between groups. Patients with severe CCM had the lowest number of cycles and longest switching time-delays, followed by patients with mild-to-moderate CCM, the age-matched control group, and the young control group. The time per cycle and the peak angular velocities of fingers in participants with severe CCM were significantly lower than those in participants with mild-to-moderate CCM; however, there were no significant differences between the control groups. The peak angular velocities of fingers were significantly lower during extension motions than during flexion motions in participants with CCM. Participants with CCM have lower peak angular velocities during finger movement. Finger extension also is impaired in participants with CCM. Abnormal finger movements and the severity of myelopathy in participants with CCM can be assessed using gyro sensors.

## Introduction

Finger motion is often impaired in patients with cervical compressive myelopathy (CCM) [1]. The abnormal finger movements in patients with CCM are termed myelopathy hand [2], and result in impaired extension of the ulnar two or three fingers and an inability to grip and release rapidly [2]; however, these characteristics of myelopathy hand have not been assessed quantitatively [3].

The grip and release test (GRT) can be used to evaluate myelopathy hand. The GRT counts the number of times that an individual can make and release a fist in 10 seconds [2]. Previous reports showed that patients with CCM achieve a reduced number of cycles during the GRT [2, 4]. While the GRT is useful to evaluate myelopathy hand, it is subject to interobserver variability. Visual observation and manual counting could not guarantee the participants’ correct finger movements, and so the results were not very accurate [3–5]. Additionally, several previous studies have reported that the number of times decreases with age [6, 7], suggesting that the GRT is not specific for CCM or age. Therefore, the number of cycles in the GRT alone is not sufficient to assess myelopathy hand and describe its characteristics. Patients with CCM cannot open or close their fingers with maximum velocity [5] and cannot switch between finger flexion and extension quickly [8]. However, quantitative measures of kinematic parameters such as peak finger velocity or time-delay to switch between finger flexion and extension have not been previously reported. The quantitative measurement of these parameters can be achieved using a small gyro sensor [9, 10]. The purpose of this study was to quantitatively evaluate the abnormal finger movements in patients with CCM and to understand the characteristics of myelopathy hand during the GRT using gyro sensors.

## Methods

### Participants

Between December 2019 and December 2020, sixty patients with CCM (CCM group), 30 age-matched healthy adults (age-matched control group), and 30 young healthy adults (young control group) were recruited via advertisements at the authors’ institutions (university or hospital) and enrolled in this study. The sample size of this study was calculated using the G*Power statistical packages (G*Power Ver. 3.1.9.2, Universität Düsseldorf, Düsseldorf, Germany) [11]. The required sample size for ANOVA (repeated measures; within-between interactions) with 80% power, α = 0.05, β = 0.20, f = 0.20 effect size, four groups, three measurements per individual, and no correlation among repeated measures was calculated as 120. Therefore, we recruited a total of 120 participants for this study. The demographic data of enrolled participants are shown in Table 1 and the inclusion/exclusion criteria is shown in Table 2.

**Table 1:** Participants’ demographic data and inventory scores.

	Control group		CCM group (n = 60)	
	Young (n = 30)	Age-matched (n = 30)	Mild-to-moderate (n = 30)	Severe (n = 30)
Sex (male / female)	12 / 18	16 / 14	17 / 13	16 / 14
Age, years	26.8 (12.0)	66.5 (10.8) *	65.8 (13.1) *	68.8 (10.8) *
Edinburgh Handedness Inventory scores	96.3 (8.5)	98.5 (5.6)	98.3 (6.5)	99.0 (4.5)
JOA scores	―	15.8 (1.2)	11.8 (1.5)	7.1 (1.7)
pain or numbness in the arms or hands (VAS)	―	0.0 (0.0)	63.1 (26.3)	64.2 (25.3)

Continuous variables are presented as mean (standard deviation).

Abbreviations: CCM = cervical compressive myelopathy; JOA = Japanese Orthopaedic Association; VAS = visual analog scale.

* Significantly older than in the young control group.

Baseline values between groups were compared using the Kruskal-Wallis test for continuous parameters and the Fisher’s exact test for categorical parameters.

**Table 2:** Inclusion/exclusion criteria.

	Control group	CCM group
Inclusion criteria	Young ・Able to give informed consent ・Age from 20 to 40 years ・Male and females	・Able to give informed consent ・Age from 40 to 80 years ・Male and females ・Patients diagnosed as CCM with motor dysfunction of the hand or presented with clinical symptoms of cervical myelopathy in the upper limbs and mechanical compression of cervical spinal cord identified by MRI
	Age-matched ・Able to give informed consent ・Age from 40 to 80 years ・Male and females	
Exclusion criteria	・With medical history of orthopedic, neurological, or spinal disorders which affect the motor or sensory functions of upper extremity, such as stroke, diabetes, rheumatoid arthritis ・With medication history which affect the numbness, pain or motor dysfunction of the upper extremity ・With significant finger deformity ・With motor dysfunction of upper extremity, such as hand clumsiness ・With sensory disturbance of upper extremity, such as pain, numbness, or paraesthesia	・Except for CCM, with medical history of orthopedic, neurological, or spinal disorders which may affect the motor or sensory functions of upper extremity, such as stroke, diabetes, rheumatoid arthritis ・With a history of cervical spine surgery ・With medication history which affect the numbness, pain or motor dysfunction of the upper extremity caused by other factors than CCM ・Without the motor dysfunction of the upper extremity (JOA motor function of upper extremity score was 4) ・ With significant finger deformity

Abbreviations: CCM = cervical compressive myelopathy; JOA = Japanese Orthopaedic Association; MRI = magnetic resonance imaging

Every CCM patient had sensory disturbance in their upper and/or lower extremity, and the diagnosis of CCM was confirmed with neurological testing and magnetic resonance imaging (MRI) by specialist spine surgeons (KN, YF, KY, NK, RO, SK, YT, TN, and YF). The compression was detected in the C2/3, C3/4, C4/5, C5/6, and /or C6/C7 level(s) on MRI (Table 3) [12, 13].

**Table 3:** Compressed levels in the Cervical Compressive Myelopathy (CCM) group.

Patient No.	Age	Sex	JOA score	Compressed level(s)
Severe CCM				
1	73	Female	7.5	C3/4, 4/5
2	64	Male	5.5	C3/4, 4/5, 5/6, 6/7
3	65	Male	4	C4/5
4	62	Male	6.5	C3/4, 4/5, 5/6
5	72	Male	8.5	C3/4, 4/5, 5/6, 6/7
6	70	Female	8.5	C3/4, 4/5, 5/6, 6/7
7	43	Female	6.5	C3/4
8	51	Female	8.5	C6/7
9	56	Female	8	C5/6
10	48	Male	8	C2/3
11	80	Male	7	C3/4
12	71	Female	4.5	C3/4, 4/5, 5/6, 6/7
13	79	Male	8.5	C2/3, 3/4, 4/5, 5/6, 6/7
14	71	Female	8.5	C3/4, 4/5, 5/6, 6/7
15	79	Female	5.5	C2/3, 3/4, 4/5, 5/6, 6/7
16	75	Male	5.5	C4/5, 5/6
17	80	Female	8	C5/6, 6/7
18	76	Male	8.5	C5/6
19	76	Male	8.5	C3/4, 5/6, 6/7
20	42	Female	4.5	C4/5
21	72	Male	1.5	C3/4, 4/5, 5/6
22	74	Female	8.5	C3/4, 4/5, 5/6
23	77	Male	7.5	C3/4
24	78	Female	7.5	C3/4, 4/5, 5/6, 6/7
25	73	Male	6.5	C5/6, 6/7
26	75	Female	8.5	C3/4, 4/5, 5/6, 6/7
27	62	Male	8	C3/4, 5/6, 6/7, 7/Th1
28	70	Male	7.5	C4/5, 5/6, 6/7
29	72	Female	8.5	C4/5, 5/6
30	78	Male	7	C3/4, 4/5, 5/6, 6/7
Mild-to-moderate CCM				
1	48	Male	9	C2/3
2	79	Female	11.5	C3/4
3	75	Male	11	C4/5, 5/6
4	78	Male	11	C4/5, 5/6
5	70	Female	14.5	C3/4, 4/5, 5/6, 6/7
6	57	Male	10.5	C5/6
7	68	Female	12	C3/4, 4/5, 5/6, 6/7
8	69	Male	11.5	C5/6
9	53	Female	14	C4/5, 5/6
10	80	Female	9.5	C3/4, 4/5, 5/6
11	60	Female	12	C3/4, 5/6
12	65	Male	13	C2/3, 3/4, 4/5, 5/6
13	76	Male	12.5	C2/3, 3/4, 4/5, 5/6, 6/7
14	78	Female	11.5	C5/6, 6/7
15	80	Male	11	C3/4, 4/5, 6/7
16	44	Male	12	C5/6
17	81	Male	15	C3/4, 4/5, 5/6
18	59	Male	14	C3/4, 4/5
19	79	Male	13	C3/4, 4/5, 6/7
20	47	Female	13.5	C2/3, 3/4, 4/5, 5/6, 6/7
21	46	Female	10.5	C5/6
22	79	Male	12.5	C2/3
23	57	Female	12	C3/4, 4/5, 5/6, 6,7
24	74	Female	10.5	C3/4, 4/5, 5/6, 6/7
25	80	Female	13.5	C4/5, 5/6, 6/7
26	70	Female	9.5	C3/4, 4/5, 5/6
27	49	Male	11.5	C4/5
28	54	Male	11	C3/4, 4/5, 5/6
29	43	Male	11	C3/4, 4/5, 5/6, 6/7
30	77	Male	10.5	C4/5, 5/6, 6/7

Abbreviations: CCM = cervical compressive myelopathy; JOA = Japanese Orthopaedic Association

The CCM group was divided into two subgroups (severe CCM and mild-to-moderate CCM groups) based on the Japanese Orthopaedic Association (JOA) score. The JOA score is an objective evaluation scale that assesses the clinical severity of cervical myelopathy [14]. The JOA score ranges from -2 to 17, with higher scores indicating a better condition (S1 Table). JOA scores were determined for all individuals in the CCM and the age-matched control groups. Based on a previous study [15], patients in the CCM group were classified as severe CCM if the JOA score was < 9 points (n = 30) and mild-to-moderate CCM if the score was ≥ 9 points (n = 30) (Table 1). Furthermore, pain or numbness in the arms or hands was evaluated using the visual analog scale (VAS). VAS scores range from 0 to 100, with higher scores indicating a worse condition. VAS scores were also determined for all individuals in the CCM and age-matched control groups.

The control volunteers were recruited via advertisement at the authors’ institutions and enrolled as asymptomatic subjects in this study.

All participants were classified as consistent right-handers using the Edinburgh inventory test [16].

The purpose of the study and the procedures used were explained to all participants, and written informed consent was obtained. This study was approved by Ethics Committee for Epidemiology of Hiroshima University (approval number: E-2009) and performed in accordance with the Declaration of Helsinki.

### Grip and release test

The GRT is often conducted for 10 seconds [2, 6, 7, 17], though some studies expanded the test time [4, 5, 18–21]. As the 10-second GRT has not been validated the reliability [5] and the time extended GRT (15-second GRT) has also been insufficient to understand the characteristics of myelopathy hand, we used a 30-second GRT in this study. The results were evaluated to determine if a 10-second GRT is sufficient as a clinical assessment.

**Fig 1:**
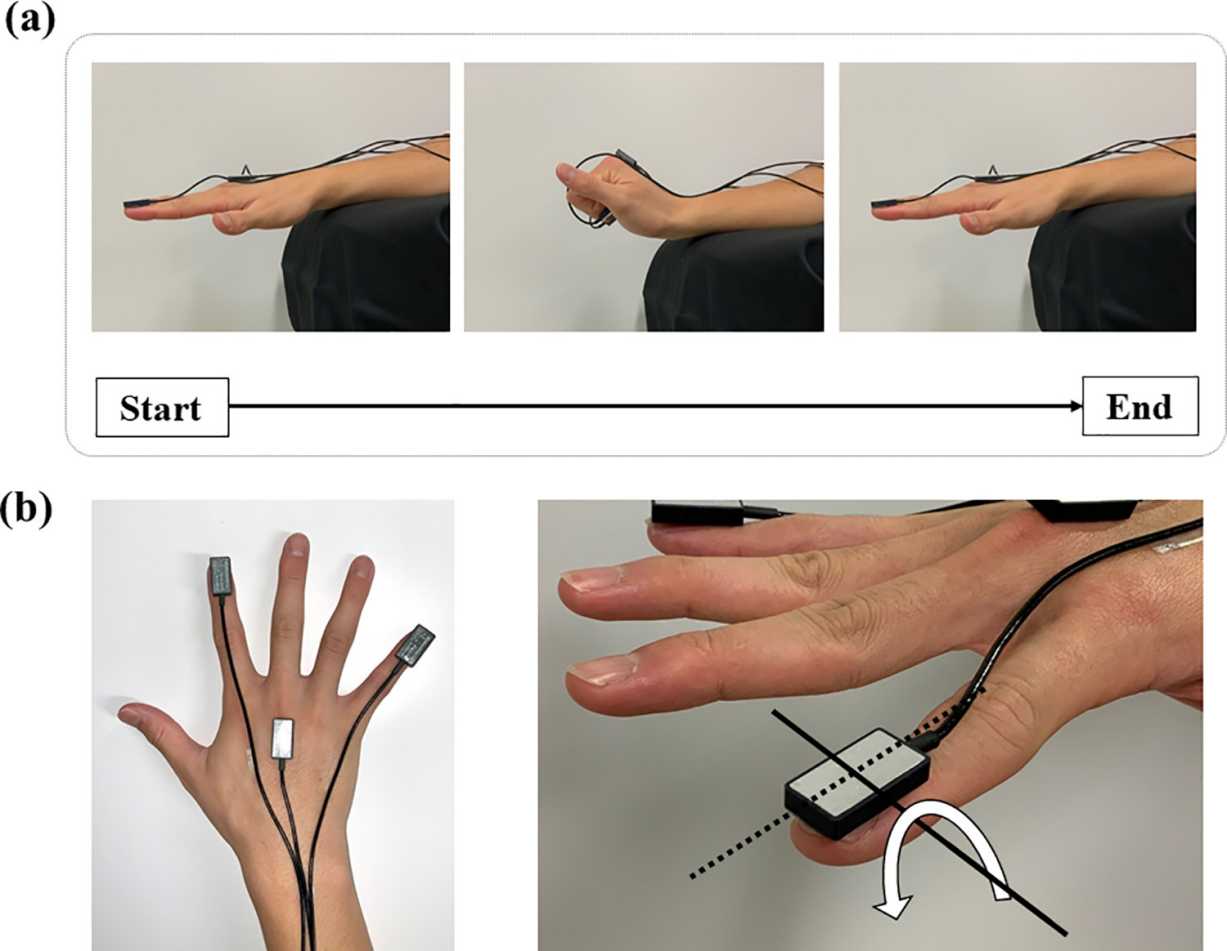
The grip and release motion during measurement, and the fixed positions of the sensors. (a) A grip and release cycle (extension to full flexion to extension) is shown. (b) The left panel shows the position of the sensors on the index and little fingers and the third metacarpal head. The right panel shows the direction of the angular velocity around the lateral-axis of the sensor.

All participants were asked to fully grip and release their right- or left-hand as fast as possible for 30 seconds. The forearm placed on a stable armrest in pronation to minimize the influence of the forearm motions (Fig 1A). The GRT was conducted twice on each limb.

### Apparatus and procedures

Three triaxial gyro sensors (MP-M6-06/2000C, size 12 mm in width; 23 mm in depth; 5 mm in height, weight 3 g, MicroStone, Nagano, Japan) were attached to the surface of the nails of the index and little fingers and the third metacarpal head using double-sided adhesive tape (Fig 1B, left). The sensor cords were fixed with tape after ensuring that their placement did not affect the finger movements. The angular velocities of the fingers and the wrist were measured from these sensors, respectively. The sampling rate was 100 Hz. The angular velocities around the lateral-axis of the sensors were used for the data analysis in this study (Fig 1B, right).

### Data analysis

**Fig 2:**
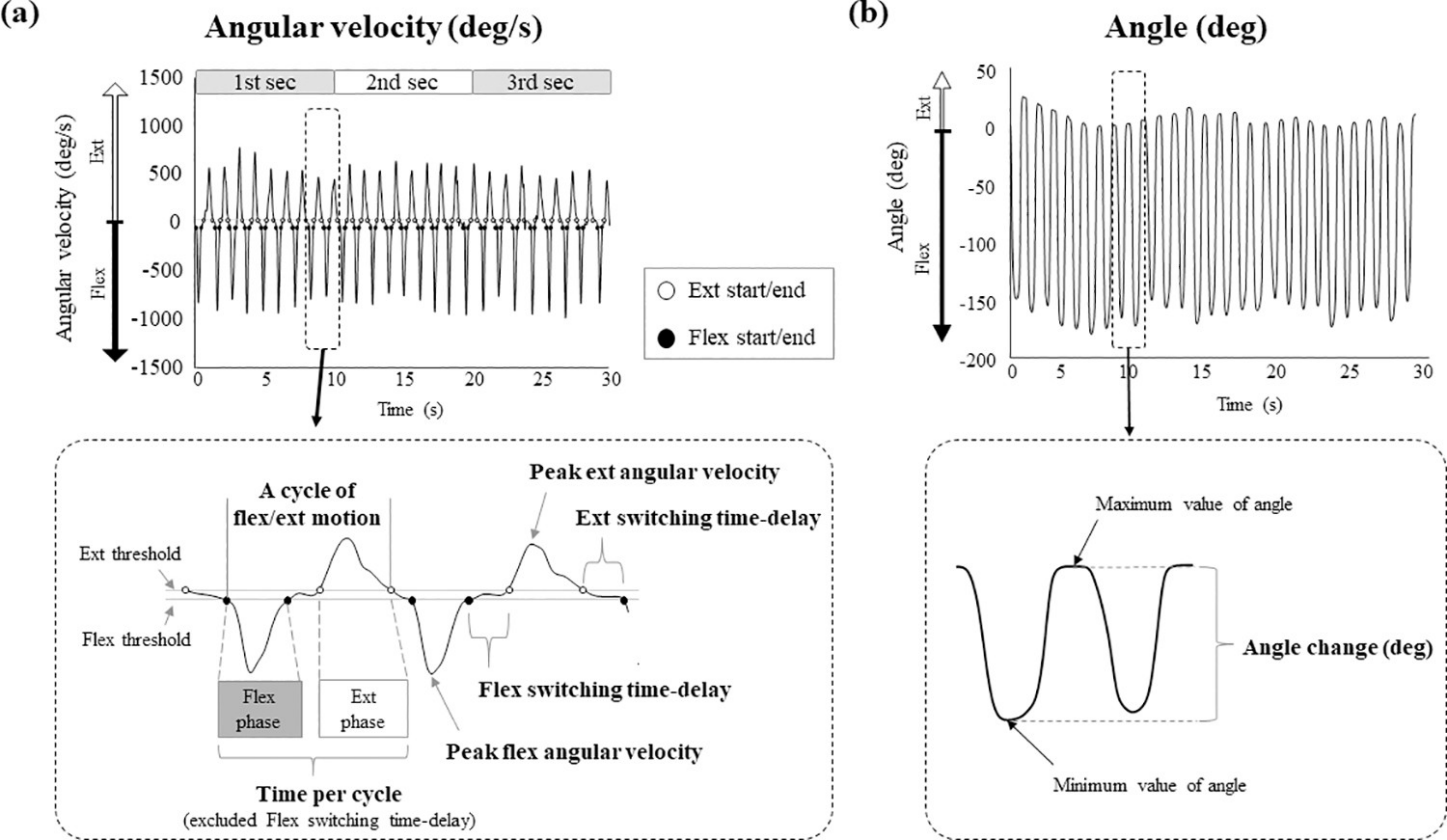
The detection methods of the kinematic parameters. (a) The upper panel shows a typical waveform of angular velocity of the index finger in a patient with cervical compressive myelopathy. The lower panel includes an illustration of the detection methods of the number of cycles, the switching time-delay, the time per cycle, and the peak angular velocity. (b) The upper figure shows a waveform of the angle in the same patient shown in Fig 2A. The lower panel includes an illustration of the detection method of the angle change. Abbreviations: Deg- degree; Ext- extension; Flex- flexion; Sec- section.

MATLAB R2019a (MathWorks Inc., USA) was used for data processing in this study. The angular velocity was filtered using a low-pass filter with a cutoff frequency of 10 Hz. A typical waveform is shown in Fig 2A. Each waveform was separated into flexion and extension phases based on the angular velocities of the fingers. The flexion/extension start/end thresholds were defined as 5% of the mean minimum or maximum angular velocities during the trial [22]. Preliminary statistical analysis using a one-way analysis of variance (ANOVA) between the three sensors showed no significant differences between the sensors’ start/end times of flexion and extension, and therefore the waveforms of the index and little fingers, as well as the wrist, were approximately synchronized. The following parameters were calculated: number of cycles (from flexion start to extension end), time per cycle (the time from the start of flexion to the end of extension, excluding the flexion switching time-delay), switching time-delay of finger, peak angular velocity of finger, and angle change of finger and wrist (Fig 2). To exclude the influence of wrist and forearm motions, other waveforms were created by subtracting the angular velocity of the wrist sensor from the angular velocity of the finger sensors, and calculating the peak angular velocity and switching time-delay based on the generated waveforms. The time from the end of flexion to the start of extension was defined as the flexion switching time-delay, and the time from the end of extension to the start of flexion was defined as the extension switching time-delay. The minimum value (negative value) of angular velocity during the flexion phase was regarded as the peak flexion angular velocity, while the maximum value of angular velocity during the extension phase was regarded as the peak extension angular velocity (Fig 2A). The difference between the maximum and the minimum angle from the start of flexion to the end of extension was defined as the angle change (Fig 2B) [9].

The number of cycles, the switching time-delay, and the peak angular velocity were also determined separately for the first, second, and third 10-second periods of the 30-second GRT.

### Statistical analysis

All data are presented as the mean and standard deviation (SD) of the four trials (two trials using each limb) of the GRT. SPSS version 23 statistical software (IBM Inc., Chicago, IL, USA) was used for statistical analysis. Statistical significance was set at a p < 0.05.

The demographic data between groups were compared using the Kruskal-Wallis test for continuous parameters and the Fisher’s exact test for categorical parameters. The JOA and VAS scores were compared using ANOVA with a factor of group (severe CCM, mild-to-moderate CCM, and age-matched control).

The angle change and time per cycle were compared using a one-way ANOVA for group (severe CCM, mild-to-moderate CCM, age-matched control, and young control). The number of cycles and switching time-delay were compared using a two-way ANOVA for group and section (first, second, and third). The peak angular velocity was compared using a three-way ANOVA for group, section, and direction (flexion and extension). Post-hoc tests were performed using a Bonferroni’s test.

## Results

### Participants

The participants’ demographic data are shown in Table 1. Age was the only significantly different demographic variable that was different between the groups, as the young control group was significantly younger than the other groups (p < 0.01). Sex and handedness scores were not statistically different between groups. JOA scores were significantly different between the groups (F = 253.6, p < 0.01). Participants with severe CCM and those with mild-to-moderate CCM had significantly lower JOA scores than participants in the age-matched control group (p < 0.01). The scores in the severe CCM group were significantly lower than those in the mild-to-moderate CCM group (p < 0.01). VAS scores were significantly different between the groups (F = 91.2, p < 0.01). Participants in the CCM groups had significantly higher VAS scores than participants in the age-matched control group (p < 0.01). There was no significant difference in VAS scores between the CCM groups.

### Measurement data

**Fig 3:**
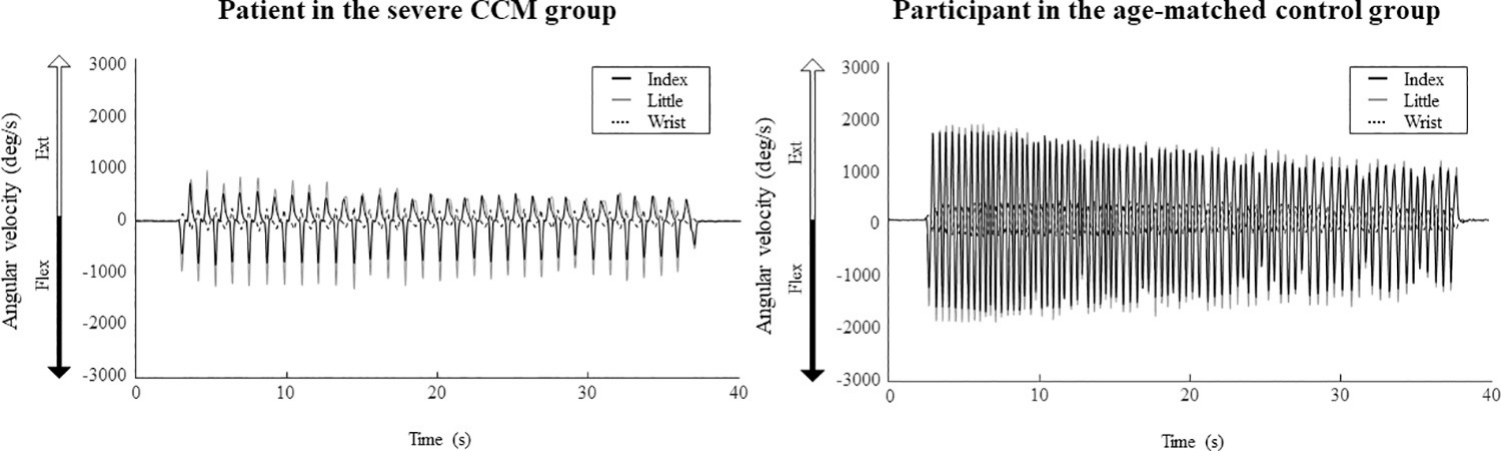
Typical waveforms of the angular velocity during the grip and release test in a patient in the severe CCM group and a participant in the age-matched control group. The left panel shows the angular velocity of the right fingers and wrist of a 73-year-old male patient in the severe CCM group with a JOA score of 7.5. The right panel shows the angular velocity of the right fingers and wrist of a 72-year-old female participant in the age-matched control group with a JOA score of 17. Abbreviations: CCM- cervical compressive myelopathy; Deg- degree; Ext- extension; Flex- flexion; JOA- Japanese Orthopaedic Association.

Representative waveforms of the angular velocity during the GRT of a patient in the severe CCM group and a participant in the age-matched control group are shown in Fig 3. The waveforms of the flexion and extension of the fingers and wrist are opposite due to tenodesis-action. Some kinematic parameters such as the number of cycles and the amplitude of the angular velocity of a patient with severe CCM group are lower than those of a participant in the age-matched control group.

#### Angle change

The finger and wrist angle changes were not significantly different between the groups (index finger: F = 1.3, p = 0.286; little finger: F = 0.2, p = 0.907; wrist: F = 0.9, p = 0.469) (Table 4).

**Table 4:** Angle change.

	Control group		CCM group	
	Young	Age-matched	Mild-to-moderate	Severe
Index	169.6 (13.1)	169.2 (11.7)	168.2 (11.9)	163.9 (13.7)
Little	165.8 (14.3)	168.2 (21.6)	168.5 (23.6)	169.6 (20.8)
Wrist	32.7 (7.6)	33.3 (8.4)	31.0 (12.8)	30.0 (5.6)

Variables are presented as mean (standard deviation).

Abbreviations: CCM = cervical compressive myelopathy; Index = index finger; Little = little finger.

#### Number of cycles

The number of cycles was significantly different between groups (F = 135.6, p < 0.01), sections (F = 51.9, p < 0.01), and the group × section interaction (F = 11.9, p < 0.01) (Table 5 and S1 Fig). Participants in the severe CCM group completed the lowest number of cycles, followed by those in the mild-to-moderate CCM group and those in the age-matched control group. Participants in the young control group completed the highest number of cycles (Table 5). In the young control group, the number of cycles in the second section was significantly lower than that in the first section (p < 0.05). The number of cycles in the third section was significantly lower than that in the first and second sections in the young control and age-matched control groups (p < 0.05). There was no significant difference between sections in the CCM groups (S1 Fig).

**Table 5:** Kinematic parameters during the GRT.

				Control group		CCM group	
				Young	Age-matched	Mild-to-moderate	Severe
**Number of cycles**			Total	90.7 (15.8)	71.3 (14.5) ^a^	53.8 (9.9) ^a^^,^^b^	30.8 (8.4) ^a,b,c^
			1st	32.6 (6.3)	25.0 (5.1) ^a^	18.2 (3.8) ^a^^,^^b^	10.5 (2.8) ^a,b,c^
			2nd	31.2 (5.2)	24.7 (5.0) ^a^	18.0 (3.4) ^a^^,^^b^	10.4 (2.9) ^a,b,c^
			3rd	26.8 (5.0)	21.5 (4.8) ^a^	17.6 (3.1) ^a^^,^^b^	9.9 (3.0) ^a,b,c^
**Switching time-delay (ms)**	Flex	Index	Total	31.0 (15.7)	50.8 (13.5) ^a^	95.2 (22.1) ^a^^,^^b^	146.8 (24.1) ^a,b,c^
			1st	27.7 (14.4)	49.5 (17.4) ^a^	92.2 (23.0) ^a^^,^^b^	134.9 (32.5) ^a,b,c^
			2nd	31.0 (15.5)	49.8 (12.7) ^a^	93.8 (22.7) ^a^^,^^b^	144.1 (20.8) ^a,b,c^
			3rd	35.3 (19.4)	55.6 (14.5) ^a^	100.5 (26.4) ^a^^,^^b^	161.9 (32.9) ^a,b,c^
		Little	Total	47.1 (19.1)	71.9 (14.4) ^a^	89.5 (34.8) ^a,b,^	156.6 (55.4) ^a,b,c^
			1st	44.3 (16.3)	68.6 (17.5) ^a^	88.8 (29.0) ^a^	151.4 (55.6) ^a,b,c^
			2nd	46.7 (19.6)	71.1 (14.6) ^a^	94.0 (31.8) ^a^^,^^b^	147.0 (50.2) ^a,b,c^
			3rd	50.3 (24.1)	77.8 (18.2) ^a^	99.5 (32.4) ^a^	172.3 (65.9) ^a,b,c^
	Ext	Index	Total	27.4 (18.9)	49.0 (20.2) ^a^	99.6 (37.8) ^a^^,^^b^	149.2 (37.9) ^a,b,c^
			1st	23.4 (12.0)	49.5 (24.5) ^a^	103.7 (44.6) ^a^^,^^b^	144.0 (32.2) ^a,b,c^
			2nd	28.7 (23.1)	48.6 (20.7)	99.5 (40.2) ^a^^,^^b^	147.6 (44.4) ^a,b,c^
			3rd	30.3 (23.3)	49.7 (17.6)	101.8 (29.6) ^a^^,^^b^	146.8 (40.9) ^a,b,c^
		Little	Total	24.9 (22.8)	47.3 (24.1)	107.7 (56.2) ^a^^,^^b^	158.0 (34.9) ^a,b,c^
			1st	22.8 (17.8)	46.1 (24.1)	103.1 (57.7) ^a^^,^^b^	151.8 (41.9) ^a,b,c^
			2nd	24.9 (24.8)	48.1 (28.3)	100.1 (55.5) ^a^^,^^b^	164.7 (38.8) ^a,b,c^
			3rd	27.5 (28.7)	49.0 (25.2)	94.0 (39.8) ^a^^,^^b^	157.0 (40.6) ^a,b,c^
**Peak angular velocity (deg/s)**	Flex	Index	Total	1348.3 (153.4)	1301.9 (150.2)	1019.4 (141.7) ^a^^,^^b^	782.5 (207.3) ^a,b,c^
			1st	1447.9 (179.1)	1395.5 (184.8)	1076.3 (136.2) ^a^^,^^b^	816.1 (233.9) ^a,b,c^
			2nd	1343.2 (166.2)	1297.2 (153.7)	1007.3 (151.2) ^a^^,^^b^	781.8 (221.0) ^a,b,c^
			3rd	1242.9 (170.5)	1199 (154.9)	977.1 (163.7) ^a^^,^^b^	758.8 (190.4) ^a,b,c^
		Little	Total	1450.1 (222.2)	1398.8 (199.9)	1198.9 (230.3) ^a^^,^^b^	865.2 (203.5) ^a,b,c^
			1st	1529.9 (232.8)	1485 (224.6)	1266.0 (243.9) ^a^^,^^b^	891.0 (208.1) ^a,b,c^
			2nd	1450 (238.7)	1381.8 (223.6)	1188.6 (241.8) ^a^^,^^b^	859.2 (204.0) ^a,b,c^
			3rd	1357.2 (220.1)	1316.9 (196.5)	1143.6 (229.9) ^a^^,^^b^	833.8 (204.1) ^a,b,c^
	Ext	Index	Total	1354.5 (173.3)	1294.3 (172.1)	935.2 (198.2) ^a^^,^^b^	660.9 (191.6) ^a,b,c^^,^*
			1st	1450 (198.4)	1419.3 (186.0)	1008.8 (210.8) ^a^^,^^b^	696.5 (211.7) ^a,b,c^^,^*
			2nd	1347.8 (196.0)	1280 (174.2)	945.0 (203.4) ^a^^,^^b^	611.1 (202.4) ^a,b,c^^,^*
			3rd	1257.2 (192.6)	1168.1 (188.3)	900.6 (197.6) ^a^^,^^b^	601.4 (184.5) ^a,b,c^^,^*
		Little	Total	1429.9 (198.2)	1352.2 (197.4)	1120.4 (226.5) ^a^^,^^b^	688.5 (180.2) ^a,b,c^^,^*
			1st	1484.7 (223.5)	1445.6 (207.4)	1196.4 (233.0) *	717.9 (207.2) ^a,b,c^^,^*
			2nd	1431 (216.3)	1337.3 (213.9)	1119.4 (247.2) *	687.3 (191.1) ^a,b,c^^,^*
			3rd	1369.3 (184.9)	1262.9 (203.9)	1073.6 (230.7) *	638.1 (217.0) ^a,b,c^^,^*

Data are presented as mean (standard deviation).

Abbreviations: CCM = cervical compressive myelopathy; Flex = flexion, Ext = extension, Index = index finger, GRT = grip and release test; Little = little finger.

^a^, ^b^, and ^c^ indicate the significant differences between groups.

^a^ Significantly lower (or longer) than that in the young control group.

^b^ Significantly lower (or longer) than that in the age-matched control group.

^c^ Significantly lower (or longer) than that in the mild-to-moderate CCM group.

* Significantly lower than the peak flexion angular velocity within the group.

#### Switching time-delay

The switching time-delays of the index and litter fingers were significantly different between groups (index finger: F = 200.6, p < 0.01; little finger: F = 117.3, p < 0.01) and sections (index finger: F = 13.3, p < 0.01; little finger: F = 8.4, p < 0.01) (Table 5 and S2 Fig).

The switching time-delay was significantly longer in the severe CCM group than in the other groups in all conditions (p < 0.01), as shown in Table 5. The switching time-delay was significantly longer in the mild-to-moderate CCM group than in the two control groups in all conditions (p < 0.05), except for little finger flexion in the first and third sections of the age-matched control group. The switching time-delay was significantly longer in the age-matched control group than in the young control group under several conditions (Table 5).

Participants in all groups had a significant prolongation of switching time-delay over the course of the 30-second GRT in some conditions (S2 Fig).

#### Time per cycle

The times per cycle of the index finger and little finger were significantly different between the groups (index finger: F = 56.6, p < 0.01; little finger: F = 63.1, p < 0.01).

The times per cycle of the index finger and the little finger were significantly longer in the severe CCM group than in the other groups (p < 0.01). The times per cycle of the index finger and the little finger were significantly longer in the mild-to-moderate CCM group than in the control groups (p < 0.01). The times per cycle of the index finger and the little finger were not significantly different between the two control groups (S3 Fig).

#### Peak angular velocity

The peak angular velocities differed between groups (index finger: F = 67.9, p < 0.01; little finger: F = 94.2, p < 0.01), directions (index finger: F = 53.1, p < 0.01; little finger: F = 33.1, p < 0.01), sections (index finger: F = 86.5, p < 0.01; little finger: F = 121.1, p < 0.01), interactions of group × direction (index finger: F = 11.0, p < 0.01; little finger: F = 13.9, p < 0.01), group × section (index finger: F = 2.9, p < 0.01; little finger: F = 6.9, p < 0.01), and group × section × direction (index finger: F = 1.6, p < 0.05; little finger F = 2.7, p < 0.05) (Table 5 and S4 Fig).

The peak angular velocity in the severe CCM group was significantly lower than that in the other groups in all conditions, as shown in Table 5. The peak angular velocity in the mild-to-moderate CCM group was also significantly lower than that in both control groups in all conditions. There were no significant differences in the peak angular velocity between the control groups in any condition. In the CCM groups, the peak extension angular velocities were significantly lower than the peak flexion angular velocities in both fingers (p < 0.05) (Table 5).

The peak angular velocity decreased over the course of the 30-second GRT in all conditions of both control groups, while the peak angular velocity did not decrease over the course of the 30-second GRT in some conditions of the CCM groups (S4 Fig).

## Discussion

Most previous studies have described the characteristics of myelopathy hand qualitatively; however, in this study, we used gyro sensors to assess the characteristics quantitatively. Participants with CCM completed fewer cycles, had longer time per cycle and switching time-delay measurements, and lower peak angular velocity than participants without CCM. We found that the angular velocity reflected the severity of myelopathy regardless of age. In contrast angle changes of the fingers and the wrist were not significantly different between the groups, suggesting that participants could fully grip and release a fist during the GRT in this study.

The number of cycles completed decreased with both myelopathy and age. These results are consistent with those of previous reports [6, 7, 23]. However, these reports did not clearly indicate the reason for the decrease in the number of cycles. We found that the switching time-delay was affected by the severity of myelopathy and participant age, whereas the time per cycle was not affected by age, suggesting that the decrease in the number of cycles with age may be the result of long switching time-delays.

Peak angular velocity was lowest in the severe CCM group, followed by the mild-to-moderate CCM group and the control groups. There was no significant difference in peak angular velocity between the control groups. These results suggest that the peak angular velocity decreased with the severity of myelopathy regardless of age. Previous studies have reported that patients with CCM cannot open or close their fingers quickly due to pyramidal tract disorders, secondary to cervical compression [2, 5]. Peak angular velocity reflects pyramidal tract disorders. The measurement of peak angular velocity is useful as it is not affected by age, unlike the number of cycles evaluated using conventional GRT. Furthermore, the peak extension angular velocities of both index and little fingers were significantly lower than the peak flexion angular velocities in the CCM groups in this study. Patients with CCM are characterized by an inability to extend their ulnar two or three fingers [2]. However, recent studies have revealed that the abnormal finger movements in patients with CCM also occur in the radial fingers, including the index finger [3, 24, 25]. Therefore, the significantly lower peak extension angular velocities of both fingers, observed in this study, were consistent with recent studies and could be considered characteristics of myelopathy hand in patients with CCM.

By contrast, the number of cycles, the switching time-delay, and the peak angular velocity in the severe CCM group did not significantly deteriorate over the course of the 30-second GRT under almost all conditions. This may be because patients with severe CCM are able to open or close their fingers slowly, as though their fingers are frozen (termed the frozen phenomenon) [5]. However, in the control group, the abovementioned kinematic parameters worsened during the 30-second GRT. This may be due to fatigue caused by the fact that participants need to exert maximum effort to grip and release their fingers as fast as possible (termed the fatigue phenomenon) [5]. Therefore, the severe CCM group might be less affected by fatigue because the freezing phenomenon only allowed them to slowly move their fingers. Moreover, the kinematic parameters in the CCM groups were lower than those in the control groups in the first section, indicating that a 10-second GRT is sufficient to clinically assess myelopathy hand.

One recent study quantitatively described the characteristics of myelopathy using hand gloves with bend sensors [3]. In this previous study, the number of cycles, range of motion of the fingers, and time per cycle were measured, and the authors reported that patients with CCM had fewer cycles and a longer time per cycle than healthy participants. Our study added two more kinematic parameters, namely angular velocity and switching time-delay, to the abovementioned parameters. Our results showed that patients with CCM not only had fewer cycles and a longer time per cycle, but also a lower angular velocity and a longer switching time-delay, indicating that the characteristics of myelopathy hand were revealed in more detail in our study.

The clinical applications of gyro sensors are increasing [10]. The methods used in this study to measure the kinematic parameters provide visual feedback regarding finger impairment of patients with CCM, as shown in Fig 3. Additionally, we provide that patient data can be stored and compared to their future performance, allowing for objective comparisons of a patient’s deterioration or improvement.

This study is not without limitations. First, the MRI was not performed on participants in the control group. Previous study using MRI showed that the prevalence of cervical cord compression (CCC), which is an early stage of the disease before myelopathic signs appear, is 24.4% in elderly people [26]. Although we carefully recruited the participants based on the inclusion/exclusion criteria, some participants in the control group might potentially have CCC. In future studies, we will examine and compare the finger movements between individuals with CCC and those verified to be without CCC. Second, different levels of spinal cord damage due to compression may possibly affect finger movement. However, since most of the CCM patients in this study had multi-level compression of the cervical spine, it was difficult to identify the responsible level of spinal cord injury. Therefore, it was not possible to separate the subgroups according to the affected spinal level to show the differences in finger movements. Additionally, we did not compare the parameters between the index and little fingers due to the differences in the moment arm of the fingers. The smaller moment arm of the little finger generally results in a larger angular velocity. The cross-sectional design also limits this study; therefore, a longitudinal investigation should be conducted in the future.

## Conclusion

Patients with CCM have lower peak angular velocity of their fingers and finger extension impairment is a characteristic of severe CCM. Abnormal finger movements and the severity of myelopathy in participants with CCM can be quantitatively assessed using gyro sensors.

## Supporting information

S1 Fig Number of cycles. The number of cycles in each section of each group is shown. * Significantly lower than in the first 10 seconds. † Significantly lower than in the second 10 seconds. Abbreviations: CCM = cervical compressive myelopathy.
(TIF)

S2 Fig Switching time-delay. The switching time-delays of the index finger and little finger are shown for each group during each 10-second section of the grip and release test. * Significantly longer than in the first 10 seconds. † Significantly longer than in the second 10 seconds. Abbreviations: CCM = cervical compressive myelopathy.
(TIF)

S3 Fig Time per cycle. The time per cycle of the index finger and little finger is shown for each group. * Significantly longer than in the young control group. † Significantly longer than in the age-matched control group. ‡ Significantly longer than in the mild to moderate group.
(TIF)

S4 Fig Peak angular velocity. The peak angular velocities of the index finger and little finger are shown for each group during each 10-second section of the grip and release test. * Significantly lower than in the first 10 seconds. † Significantly lower than in the second 10 seconds. Abbreviations: CCM = cervical compressive myelopathy.
(TIF)

S1 Table Evaluation of the severity of myelopathy using the JOA score. (DOCX)
