# Supplementary figures and images for: Quantitative evaluation of abnormal finger movements in myelopathy hand during the grip and release test using gyro sensors
Source: PLoS One. 2021 Oct 20;16(10):e0258808. doi: 10.1371/journal.pone.0258808 (PMC8528295; doi:10.1371/journal.pone.0258808)

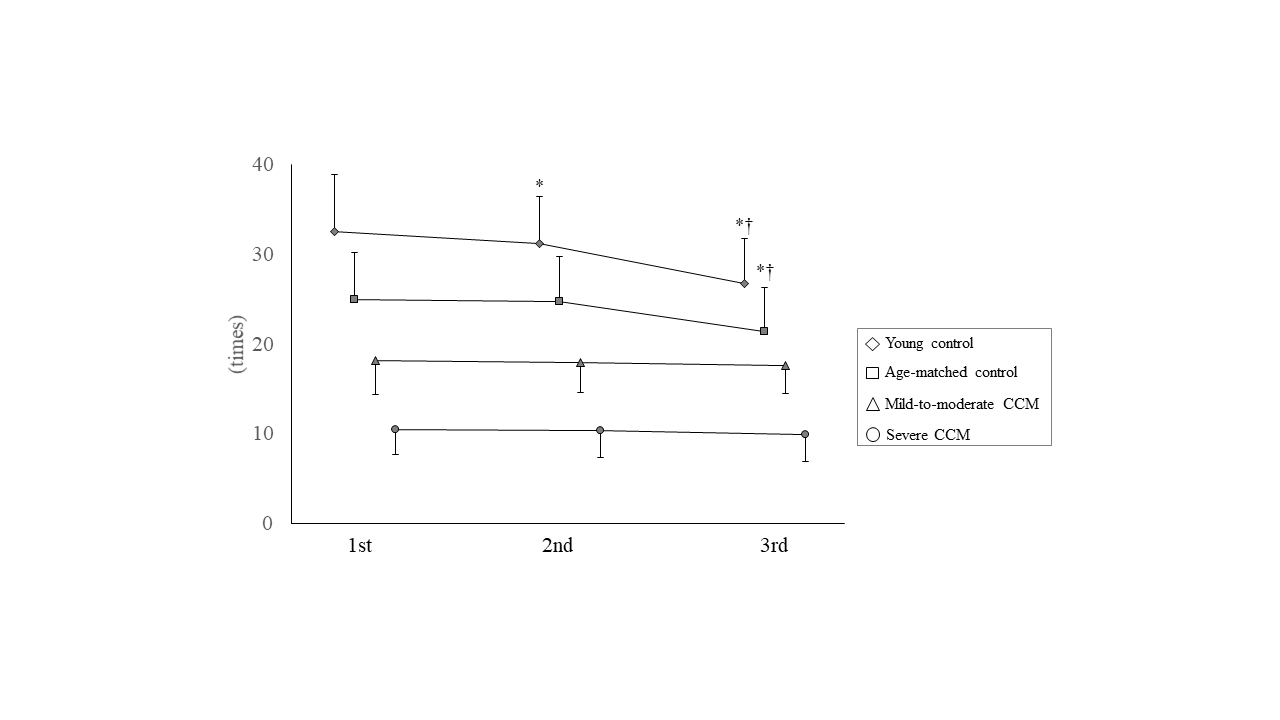

Supplement: S1 Fig — The number of cycles in each section of each group is shown. * Significantly lower than in the first 10 seconds. † Significantly lower than in the second 10 seconds. Abbreviations: CCM = cervical compressive myelopathy. (TIF) [file pone.0258808.s001.tif]

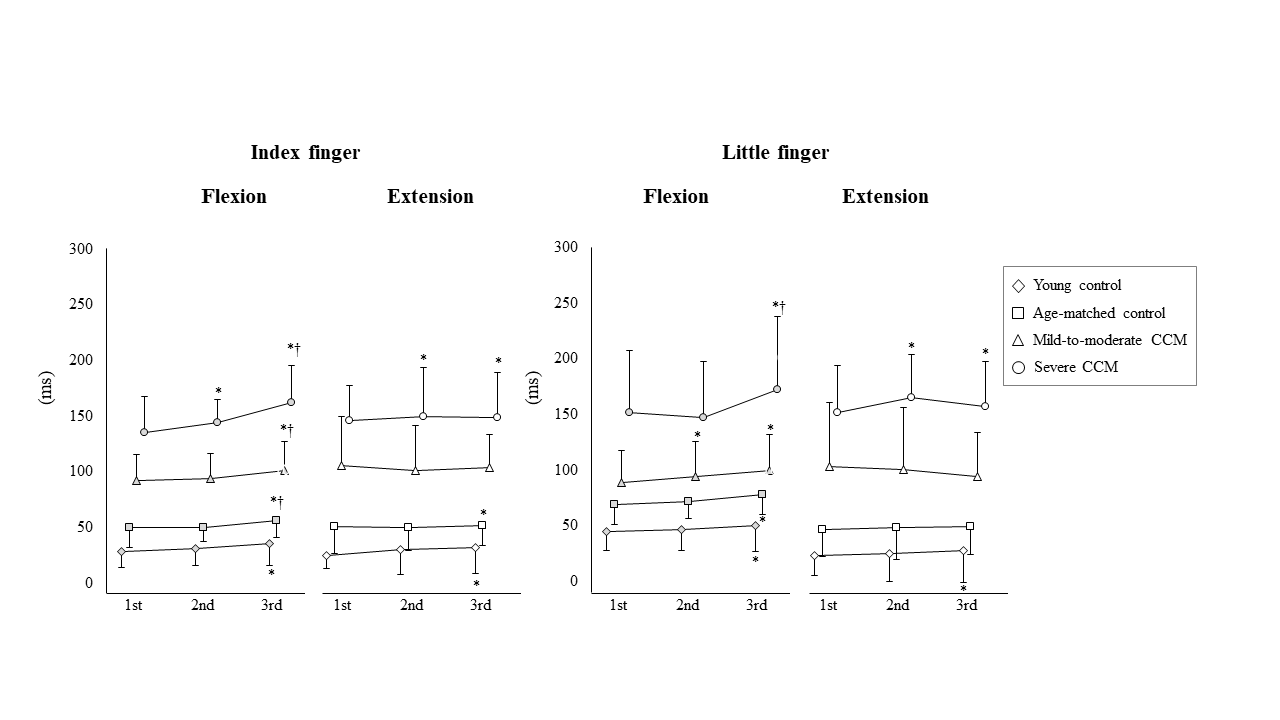

Supplement: S2 Fig — The switching time-delays of the index finger and little finger are shown for each group during each 10-second section of the grip and release test. * Significantly longer than in the first 10 seconds. † Significantly longer than in the second 10 seconds. Abbreviations: CCM = cervical compressive myelopathy. (TIF) [file pone.0258808.s002.tif]

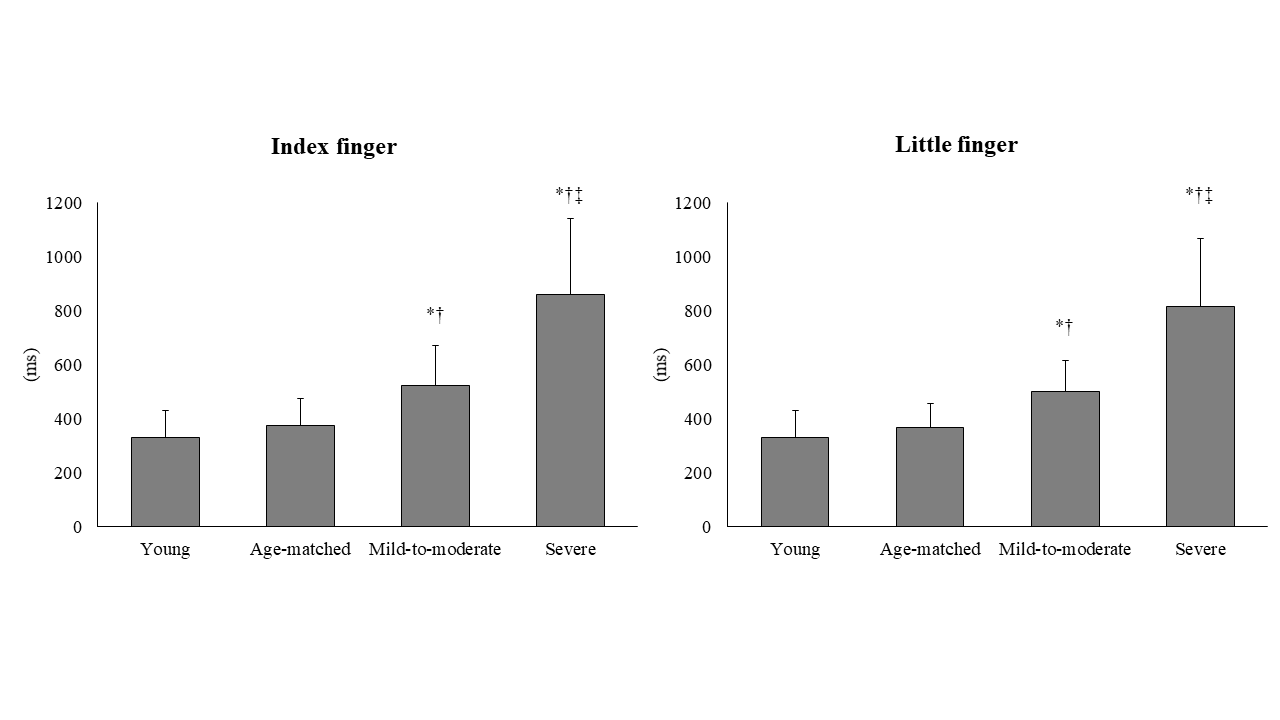

Supplement: S3 Fig — The time per cycle of the index finger and little finger is shown for each group. * Significantly longer than in the young control group. † Significantly longer than in the age-matched control group. ‡ Significantly longer than in the mild to moderate group. (TIF) [file pone.0258808.s003.tif]

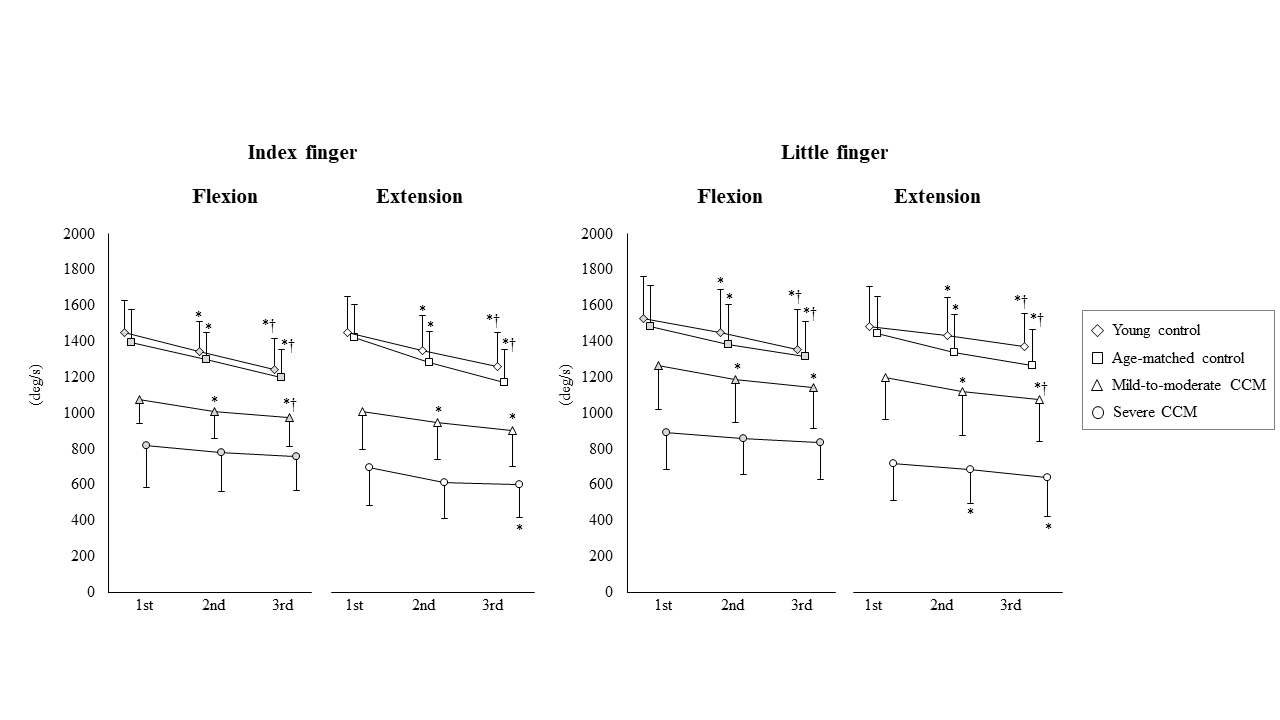

Supplement: S4 Fig — The peak angular velocities of the index finger and little finger are shown for each group during each 10-second section of the grip and release test. * Significantly lower than in the first 10 seconds. † Significantly lower than in the second 10 seconds. Abbreviations: CCM = cervical compressive myelopathy. (TIF) [file pone.0258808.s004.tif]
