# Supplementary material for: Quantitative evaluation of abnormal finger movements in myelopathy hand during the grip and release test using gyro sensors
Source: PLoS One. 2021 Oct 20;16(10):e0258808. doi: 10.1371/journal.pone.0258808 (PMC8528295; doi:10.1371/journal.pone.0258808)
Supplement: S1 Table — (DOCX) [file pone.0258808.s005.docx]

**S1 Table. Evaluation of the severity of myelopathy using the JOA score**

| JOA score |
| --- |
| Ⅰ. Motor function |
| Fingers |
| 0 Unable to feed oneself with any tableware including chopsticks, spoon, or fork, and/or unable to fasten buttons of any size  1 Can manage to feed oneself with a spoon and/or fork but not with chopsticks  2 Either chopstick-feeding or writing is possible but not practical, and/or large buttons can be fastened  3 Either chopstick-feeding or writing is clumsy but practical, and/or cuff buttons can be fastened  4 Normal |
| Shoulder and elbow (evaluated by MMT score of the deltoid or biceps muscles, whichever is weaker) |
| - 2 MMT 2 or less  - 1 MMT 3  - 0.5 MMT 4  0 MMT 5 |
| Lower extremity |
| 0 Unable to stand up and walk by any means  0.5 Able to stand up but unable to walk  1 Unable to walk without a cane or other support on a level  1.5 Able to walk without support but with a clumsy gait  2 Walks independently on a level but needs support on stairs  2.5 Able to walk independently when going upstairs, but needs support when going downstairs  3 Capable of fast but clumsy walking  4 Normal |
| Ⅱ Sensory function |
| Upper extremity |
| 0 Complete loss of touch and pain sensation  0.5 50% or less normal sensation and/or severe pain or numbness  1 More than 60% normal sensation and/or moderate pain or numbness  1.5 Subjective numbness of slight degree without any objective sensory deficit  2 Normal |
| Trunk |
| 0 Complete loss of touch and pain sensation  0.5 50% or less normal sensation and/or severe pain or numbness  1 More than 60% normal sensation and/or moderate pain or numbness  1.5 Subjective numbness of slight degree without any objective sensory deficit  2 Normal |
| Lower extremity |
| 0 Complete loss of touch and pain sensation  0.5 50% or less normal sensation and/or severe pain or numbness  1 More than 60% normal sensation and/or moderate pain or numbness  1.5 Subjective numbness of slight degree without any objective sensory deficit  2 Normal |
| Bladder function |
| 0 Urinary retention and/or incontinence  1 Sense of retention and/or dribbling and/or thin stream and/or incomplete continence  2 Urinary retardation and/or pollakiuria  3 Normal |
| Abbreviations: JOA = Japanese Orthopaedic Association; MMT = manual muscle test  Total for normal patient = 17 |
